# Regional differences of standardised mortality rates for ischemic heart diseases in the Slovak Republic for the period 1996–2013 in the context of income inequality

**DOI:** 10.1186/s13561-016-0099-1

**Published:** 2016-06-04

**Authors:** Beáta Gavurová, Tatiana Vagašová

**Affiliations:** Faculty of Economics, Technical University of Košice, Němcovej 32, 040 01 Košice, Slovakia

**Keywords:** Standardised mortality rate, Ischemic heart diseases, Income inequality

## Abstract

**Background:**

The aim of paper is to analyse the development of standardised mortality rates for ischemic heart diseases in relation to the income inequality in the regions of Slovakia. This paper assesses different types of income indicators, such as mean equivalised net income per household, Gini coefficient, unemployment rate, at risk of poverty threshold (60 % of national median), S80/S20 and their effect on mortality.

**Methods:**

Using data from the Slovak mortality database 1996–2013, the method of direct standardisation was applied to eliminate variances resulted from differences in age structures of the population across regions and over time. To examine the relationships between income indicators and standardised mortality rates, we used the tools of descriptive statistics and methods of correlation and regression analysis.

**Results:**

At first, we show that Slovakia has the worst values of standardised mortality rates for ischemic heart diseases in EU countries. Secondly, mortality rates are significantly higher for males compared with females. Thirdly, mortality rates are improving from Eastern Slovakia to Western Slovakia; additionally, high differences in the results of variability are seen among Slovak regions. Finally, the unemployment rate, the poverty rate and equivalent disposable income were statistically significant income indicators.

**Conclusions:**

Main contribution of paper is to demonstrate regional differences between mortality and income inequality, and to point out the long-term unsatisfactory health outcomes.

## Background

Death is defined as a biosocial feature whose risk is closely related to age, gender and health status of the population. Mortality, coupled with the fertility, represent the two key dynamic demographic processes that enter into the demographic reproduction of the population directly and affect the size, distribution, and structural composition of population [1]. The current status and the nature of mortality in Slovakia are influenced by several factors. Caselli et al. [2] distinguishe between the groups of endogenous (e.g. genetic predisposition) and exogenous factors (e.g. lifestyle). However, one of the most important features is their mutual compatibility and accumulation over the life of the populations [3].

In each country, mortality belongs to the main indicators reflecting the effectiveness of the health care system [4–7]. Increased attention is devoted to the regional disparities in mortality rates for the different causes of death abroad in relation to the demographic characteristics, particularly a socio-economic status [8–13]. Biosocial demographic characteristics include age, sex, race, while socioeconomic demographic characteristics comprise income, education, marital status, occupation, etc. [1]. Many studies show that people with lower socioeconomic status have higher level of mortality rate [14–16]. Accordingly, income mainly of elderly persons is a stronger predictor for their health than education, or marital status [17]. There is also evidence that the life expectancy at birth is smaller up to 4–6 years in groups with lower socio-economic status [18, 19].

Cheng and Kindig [20] examine differences in premature mortality between high and low income 3133 US counties. Their results show a stronger relationship of mortality and income in counties with lower incomes. In addition, Kawachi and Kennedy [21] test the relationship between six indicators of income inequality (e.g. Gini koeficient, Robin Hood index, etc.) and total mortality in the United States. They show high correlation between them, even after adjusting for taxes and transfers. Similarly, Mészáros [22] describes the evolution of mortality for the different causes of death in the context of regional differences in Slovakia, but there is no deeper analysis linked with the socioeconomic indicators of individual districts and regions.

According to National Health Information Center in Slovakia (NHIC) [23], the leading causes of death are ischemic heart diseases (IHD), codes I20-I25 by International Classification of Diseases. IHD accounts for almost 30 % of all deaths in Slovakia. Therefore, in our paper we will focuse on the analysis of IHD in the eight regions of Slovakia during the period 1996–2013, both in males and females. Ischemic heart disease is an acute or chronic cardiac dysfunction, resulting from the reduction, or cessation of oxygen and nutrients delivery to the heart muscle. The most common causes are morphological changes in the coronary arteries (coronary artery disease), usually arising from a significant reduction, or cessation, of blood flow in the developed atherosclerotic process [24]. The decline in mortality for IHD has been recorded in many countries in the world, reflecting the progress in diagnosis, treatment and prevention of coronary heart disease [25–28]. This progress leads to the increased life expectancy, improving the quality of life and functional status of patients. The underlying risk factors include elevated blood pressure, high cholesterol, obesity, smoking, alcohol, health behaviour, etc. [29]. Other important risk factors are social characteristics, affiliation to an ethnic group, as well as influence of the environment [30].

The aim of this paper is to identify the most vulnerable groups of people for the past 17 years and find regions of Slovakia with the highest IHD mortality rates, considering income inequality between them. A limitation of this study appears in unavailable data on the individual level of income in mortality database, so summary measures for income indicators in each region will be applied. The main objectives of this paper are:

- to analyse the development of IHD mortality in Slovakia in comparison with European countries;
- to reveal regional differences, both in level and development of IHD mortality in Slovakia;
- to quantify the relationship between mortality and income indicators in eight regions of Slovakia.

## Methods

Mortality rates are computed from data on the number of deaths from IHD, by sex, 5-year age groups and by individual regions of Slovakia during the period 1996–2013. Under the conditions of the contract, NHIC is the nation’s primary source of health statisitics. Data on the mid-year population at the age groups and sex in every year for individual regions were obtained from the Statistical Office of the Slovak Republic. Mortality rates were age-standardised to the revised European standard population (ESP) by age groups adopted by Eurostat (the last revision in 2012).

Mortality rates are expressed by standardised mortality rates (SMR) per 100,000 population. We used the method of direct standardisation to eliminate variances resulted from differences in age structures of the population across regions and over time, ensuring the necessary conditions for comparing regions of Slovakia. SMR are computed by the following mathematic expression:

1 $$ SMR=\frac{{\displaystyle \sum_x{m}_x\;.\;{P}_x^{*}}}{{\displaystyle \sum_x{P}_x^{*}}} $$

Where:

*x* – age/sex group 0, 1-4, 5-9, 10-14, 15-19,…, 95+

*m*_*x*_ – age-specific mortality rate (deaths per 100,000 population) in sex/age group

*P*_x_^*^– European standard population in sex/age group *x*

Standardised IHD mortality rates by sex were calculated in individual regions of Slovakia during the period 1996–2013. Analyses were conducted with the support of statistical programs MS Access and Excel version 2013.

For the study of statistical population we used methods of descriptive statistics, in particular measure of central tendency: (min, max, mean, median, mode) and measure of variability (interquartile range, standard deviation, coefficient of variation). To express the growth rate of SMR, relative differences from the average SMR in base year 1996 were calculated. Coefficient of variation was calculated by the expresion:

2 $$ CV=\frac{\operatorname{s}}{{\displaystyle \overline{x}}} $$

Where:

*s* – standard deviation

$$ \overline{x} $$ – mean

In individual regions of Slovakia we tested the relationship between SMR and following income indicators: Unemployment rate in %; Mean equivalised net income per household, EUR/month; At risk of poverty threshold (60 % of national median equivalised disposable income); S80/S20; Gini coeficient. Data on the unemployment rates were obtained from the Statistical Office of the Slovak Republic and other indicators were downloaded from EU-SILC (2013) - statistical survey on income and living conditions and poverty indicators [31, 32].

*Unemployment rate* means unemployed people as a percentage of the economically active population for the previous year.

*Mean equivalised net income per household* represents household disposable income divided by equivalent household size. Individual household members are assigned weights: 1 - the first adult household member; 0.5 - each additional adult member; 0.5 - adolescents from 14 years of age and over; 0.3 - a child younger than 14 years.

*At risk of poverty threshold (60 % of national median equivalised disposable income)* expresses the percentage of persons with an equivalent disposable income below 60 % of median national equivalent income.

*S80/S20 income quintile share ratio* is calculated as a proportion of total incomes of 20 % of the richest people in society (top quintile) relative to the total income of 20 % of the poorest people (lowest quintile).

*Gini coefficient* is an indicator of monetary poverty, showing the inequality of income distribution. “The Gini coefficient is defined as the relationship of cumulative shares of the population arranged according to the level of equivalised disposable income, to the cumulative share of the equivalised total disposable income received by them“ [32]. It can gain values from 0 (absolute income equality) to 1 (absolute income inequality).

When analysing the relationship of mortality and income indicators, we expect a positive linear relationship between mortality and these variables: unemployment rate, at risk of poverty threshold, Gini coeficient, S80/20. On the contrary, a negative linear relationship is expected of equivalent disposable income.

To examine the relationships between variables we used methods of correlation and regression analysis. By means of linear regression, we quantified the effect of individual income indicators (independent variables) on SMR (dependent variable) at a significance level of α = 0.05.

## Results and discussion

This chapter are focused on the presentation of our results which relate to the partial objectives. It is divided in the two lines. The first is devoted on the international environment, examining the development of SMR for IHD in Slovakia and EU countries. Second analytical line describes the national performance, identifying and quantifying regional differences in SMR for IHD in Slovakia, as well as the relationship between mortality and income indicators in individual regions of Slovakia.

### Development of the standardised mortality rates for ischemic heart diseases in Slovakia and selected EU countries

**Fig. 1:**
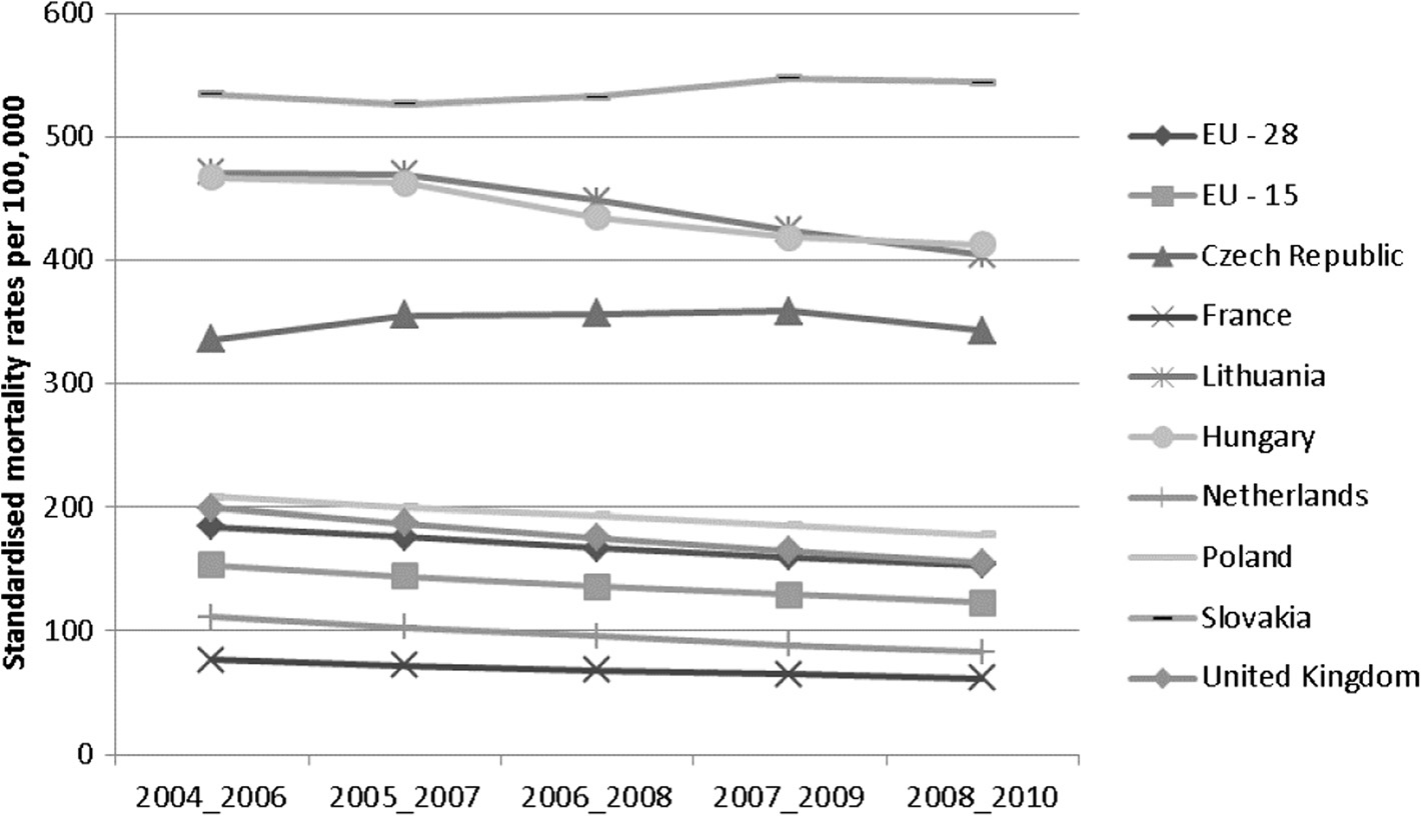
Standardised mortality rates per 100,000 population for ischemic heart diseases in the selected countries, 3 years average

Ischemic heart diseases belong to the leading causes of death in almost all EU countries. Their share in total mortality is an average of 18 % [33]. Development of SMR for IHD appeared to be slightly decreasing in selected European countries between 2004 and 2010, except for Slovakia and the Czech Republic (Fig. 1). The countries that showed the largest percentage drop in SMR were Netherlands (-25.8 %) and UK (-22.1 %). On the contrary, a rise was recorded in Slovakia (1.8 %) and in the Czech Republic (1.9 %). An average of EU-28 and EU-15 are characterised by a relatively high decrease of SMR (-16.9 %; -19.8 %). We can say that Slovakia significantly lagged behind other countries in improving of results of mortality rates in a given time span.

Based on Fig. 1 Slovakia showed the worst level of SMR (above 500), i.e., highly above the averages of EU-28 and EU-15. Values highly above the average of EU were reached in Hungary, Lithuania and Czech Republic. On the other hand, similar values as the average of EU were revealed in Poland and the United Kingdom. The Netherlands and France showed the lowest SMR, below the average of the EU-28 and EU-15. Countries of V4 region (Czech Republic, Hungary, Poland and Slovakia) presented significantly different results of SMR, suggesting variations in the effectiveness of their health systems. We expect there is a diversity of health policies approaches to reducing the incidence of risk factors, such as unhealthy lifestyles, smoking, obesity, as well as lower access to health care associated with socio-economic status population, etc. The focus of futher analyses is on the SMR for IHD in Slovakia.

**Fig. 2:**
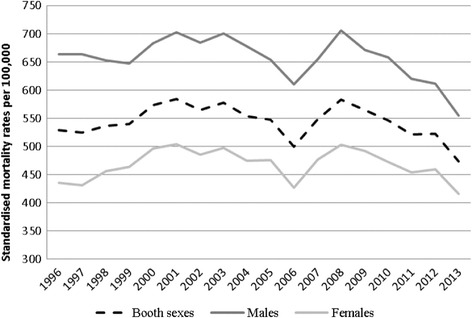
Standardised mortality rates per 100,000 population for ischemic heart diseases in Slovakia, 1996-2013, both sexes

In the long term, during the period 1996–2013, the trend of SMR was cyclical, slightly decreasing in Slovakia (Fig. 2). The SMR recorded a decrease from 663.02 in 1996 to 554.69 in 2013 (-16.34 %) for males, compared with drop from 435.06 in 1996 to 416.32 in 2013, a fall of just 4.31 % for females. Significant decrease was observed within the two periods: 2003–2006 and 2008–2011. The level of SMR for males was an average of 40 % higher compared with females. Maximum gender gaps in SMR were observed between 1996 and 1997 (53 %), while the minimum percentage difference was found in 2012 and 2013 (33 %).

**Fig. 3:**
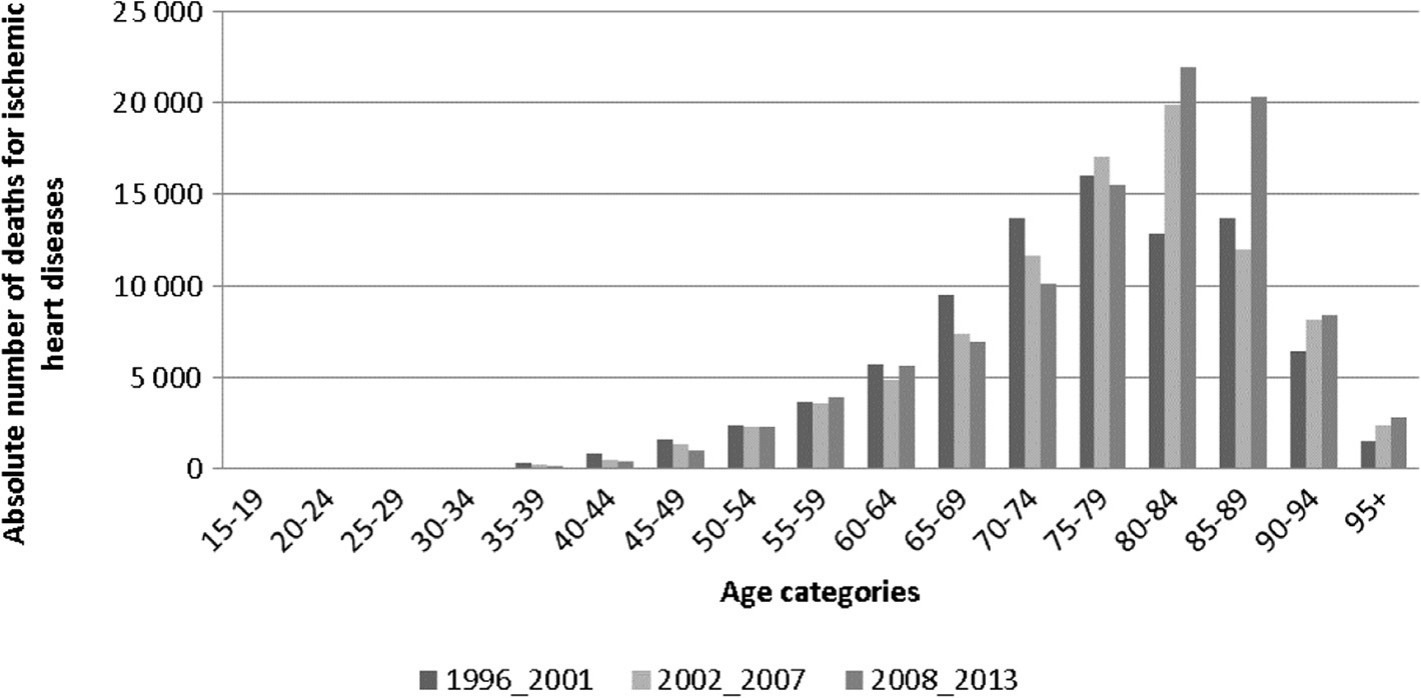
Distribution of the number of deaths for ischemic heart diseases at the age categories in Slovakia, 3 time periods

Age plays an important role in the analysis of mortality because it is a significant predictor of mortality and an indicator of at-risk age groups. In order to eliminate fluctuations of number of deaths, we divided the analysed period 1996–2013 into three phases: 1996–2001, 2002–2007 and 2008–2013. Number of deaths distribution represented by histogram (Fig. 3) reflects the observations in three different time periods in order to detect the most numerous age groups. Figure 3 shows the exponential growth in the number of deaths between age categories 15–19 and 80–84. In old age mortality, negative linear trends of frequencies are clarified. The period from 1996 to 2001 was characterised by the lowest absolute number of deaths (88,226), the most frequent age group was 75–79. In subsequent periods, age cohort 80–84 was the most frequent group and the total number of deaths increased from 91,274 to 99,512.

**Fig. 4:**
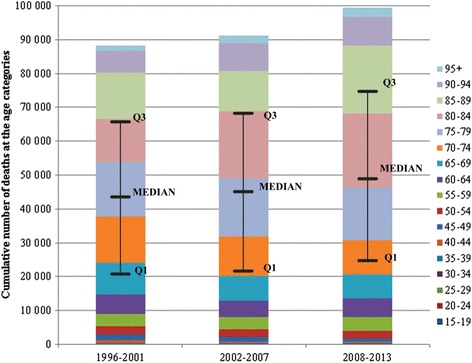
First quartile, median, third quartile of the cumulative number of deaths at the age categories in Slovakia, 3 time periods

Figure 4 shows the cumulative number of deaths at the age intervals separate in three time periods, and characterises a set of statistical data by the means of the median and interquartile range. Median was set at the age 75–79 in the first two periods then increased on the age 80–84, representing 50 % of deaths above these age groups (including). In related to the advanced age of death, we suppose that backround or origin of IHD is chronic in many cases of death. In 1996–2001 interquartile range was between age groups 65–69 and 80–84, representing age characteristics of the half of deaths. In 2002–2007, the first quartile (Q1) moved on the 70–74 age category and the third quartile (Q3) was not changed, so the 50 % share of deaths was narrowed down to the age interval 70–84. On the contrary, in the last time period the first quartile was again located at the category of 70–74 years old, and the third quartile was shifted to the age group 85–89, which indicates that the quarter died at the age 85 and older, as well as 25 % of deaths were aged 74 or less.

Our results point out the moving number of deaths to higher ages between time periods. This feature may be partly related to the ageing population in the Slovak Republic, as well as to the increase in life expectancy at birth (77.5 years in 2000; 79.9 in 2012). Therefore, in the following analysis we will consider the values of standardised mortality rate in order to eliminate the bias. For a deeper analysis of mortality for IHD in Slovakia it is desirable to examine regional differences depending on sex and long-time period.

### Regional differences of the standardised mortality rates for ischemic heart diseases

Based on The Nomenclature of Territorial Units for Statistics (NUTS III) classification, Slovakia is divided into 8 Regions: Bratislava Region (BL), Trnava Region (TA), Trenčín Region (TC), Banská Bystrica Region (BC), Košice Region (KI), Nitra Region (NI), Prešov Region (PV), Žilina Region (ZI).

In terms of individual regions of Slovakia (Table 1), between 1996 and 2001 the values of SMR ranged from 505.17 (NI) to 783.51 (KI), in 2002–2007 ranged from 522.23 (NI) to 776.44 (KI), and during the last period 2008–2013 varied from 553.22 (ZI) to 721.17 (KI) for males. During the whole time period the highest SMR were recorded in KI and the lowest in NI, as well as in ZI in the last period.

**Table 1:** Development of the standardised mortality rates per 100,000 population for ischemic heart diseases in the regions of Slovakia, 3 time periods, both sexes

Males		BC	BL	KI	NI	PV	TA	TC	ZI
1996–2001	SMR	733.81	650.51	783.51	505.17	775.34	640.40	581.64	673.43
	Rank	6.	4.	8.	1.	7.	3.	2.	5.
2002–2007	SMR	728.90	578.11	776.44	522.23	621.14	627.81	720.88	734.49
	Rank	6.	2.	8.	1.	3.	4.	5.	7.
	*Change %*	-0.67	-11.13	-0.90	*3.38*	-19.89	-1.97	*23.94*	*9.07*
2008–2013	SMR	700.92	575.77	721.17	640.99	668.25	603.88	629.67	553.22
	Rank	7.	2.	8.	5.	6.	3.	4.	1.
	*Change %*	-3.84	-0.40	-7.12	*22.74*	*7.58*	-3.81	-12.65	-24.68
	Coefficient of variantion	7.68	10.89	6.57	16.07	14.11	8.62	14.27	14.97
Females									
1996–2001	SMR	487.55	479.51	561.87	335.95	559.66	478.98	391.18	443.05
	Rank	6.	5.	8.	1.	7.	4.	2.	3.
2002–2007	SMR	506.81	444.68	556.08	361.61	447.39	458.59	505.86	512.98
	Rank	6.	2.	8.	1.	3.	4.	5.	7.
	*Change %*	*3.95*	-7.26	-1.03	*7.64*	-20.06	-4.26	*29.32*	*15.78*
2008–2013	SMR	499.39	409.44	527.46	461.24	504.05	450.06	467.66	424.48
	Rank	6.	1.	8.	4.	7.	3.	5.	2.
	*Change %*	-1.46	-7.92	-5.15	*27.55*	*12.66*	-1.86	-7.55	-17.25
	Coefficient of variantion	10.43	14.08	5.80	17.84	13.28	12.82	16.86	11.69

*SMR* Standardised Mortality Rates, *BC* Banská Bystrica Region, *BL* Bratislava Region, *KI* Košice Region, *NI* Nitra Region, *PV* Prešov Region, *TA* Trnava Region, *TC* Trenčín Region, *ZI* Žilina Region. “Rank” means that regions are ordered from 1 (the lowest SMR) to 8 (the highest SMR) in each time period. “Change %” expresses the growth rates of SMR between two time periods in each region separately. The increase is characterised by positive values, vice versa. “Coefficient of variation” presents the variability of SMR during the all periods in each region

**Fig. 5:**
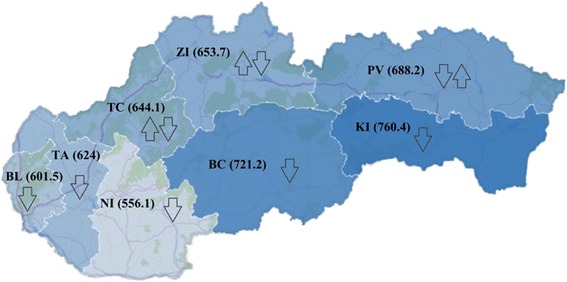
Average level and trend of the standardised mortality rates per 100,000 population for ischemic heart diseases in the regions of Slovakia, 1996-2013, males

Table 2 reflects the descriptive statistics of SMR in each time period. Between the first and second time period, median of SMR increased from 661.97 to 674.34, representing deterioration in mortality accompanied by the percentage increase of SMR between the two periods in the TC (23.94 %), ZI (9.07 %) and NI (3.38 %) (Table 1). In the third time period the median dropped to 635.33, indicating improvement in mortality. The highest percentage decreases between the periods 2002–2007 and 2008–2013 were shown in ZI (-24.68 %), TC (-12.65 %) and KI (-7.12 %). The continuing deterioration in growth rate (22.74 %) was typical for NI, and the previous improving growth rate changed into the opposite trend (7.58 %) in PV (Fig. 5). In all three time periods for males, BC and KI accounted for the level of SMR above the median, i.e., higher mortality, while BL and TA always showed the values of SMR lower than the median value, i.e., lower mortality.

**Table 2:** Descriptive statistics of the standardised mortality rates per 100,000 population for ischemich heart diseases in Slovakia, 1996–2013, both sexes

Males	1996–2001	2002–2007	2008–2013
Minimum	505.17	522.23	553.22
Maximum	783.51	776.44	721.17
Median	661.97	674.34	635.33
Standard deviation	95.82	89.19	58.65
Mean	667.98	663.75	636.74
Coefficient of variation	14.34	13.44	9.21
Females			
Minimum	335.95	361.61	409.44
Maximum	561.87	556.08	527.46
Median	479.25	482.22	464.45
Standard deviation	77.28	59.50	40.55
Mean	467.22	474.25	467.97
Coefficient of variation	16.54	12.55	8.67

Standard deviation (Table 2) expressing the rate of variability was the highest in the period 1996–2001 for males and gradually declined. Reducing variability of SMR for IHD over the period 1996–2013 is confirmed by the value of the coefficient of variation, which recorded a downward trend (Table 2). As for the dispersion of values from the average (i.e., coefficient of variation) in each region separately, according to Table 1, the highest rate of variability was reached in NI (16.07) and the lowest in KI (6.57). Although, NI was denoted as the best in terms of mortality and KI as the worst, their results of variability showed the remaining bad position in KI, conversely the high volatility of positive results in NI.

**Fig. 6:**
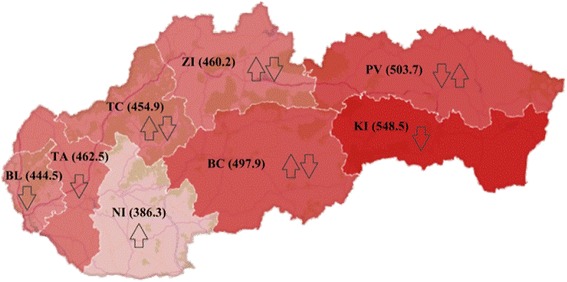
Average level and trend of the standardised mortality rates per 100,000 population for ischemic heart diseases in the regions of Slovakia, 1996-2013, females

SMR is an average of 40 % lower for females than for males. During the period 1996–2001, the values of SMR for females ranged from 335.95 (NI) to 561.87 (KI). In the period 2002–2007, a minimum value of SMR was 361.61 (NI), conversaly a maximum value was 556.08 (KI). Even in time period 2008–2013, KI kept the worst position (527.46) compared with other regions of Slovakia. The best value of SMR (409.44) was recorded in BL, while the NI fell in value of 461.24 to the fourth position among other regions (Table 1). According to the Table 1, percentage changes of SMR for females showed relatively large differences among regions. In the period 2002–2007, the highest growth rates compared to the previous time period were achieved in TC (29.32 %), ZI (15.78 %) and NI (7.64 %). Similarly, the median of SMR rose from 479.25 to 482.22 (Table 2). In the last time period, the median dropped to 464.45. This feature was accompanied by a drop in growth rate of SMR in the regions ZI (-17.25 %), BL (-7.92 %), TC (-7.55 %). The growth rate of SMR was increasing during the whole examined period in NI. Initially, PV recorded a decline (-20.06 %), then an increase (12.66 %). The opposite trend, i.e., from an increase (29.32 %) to a decline (-7.55 %), was found in TC, similarly in ZI (Fig. 6). In all three time periods for females, BC and KI accounted for the level of SMR above the median, i.e., higher mortality, while NI and TA always showed the values of SMR lower than the median value, i.e., lower mortality. Standard deviation, as well as the coefficient of variation, showed a downward trend. As well as for males, rates of variability in separate regions gained the highest values in NI (17.84) and the lowest in KI (5.8) (Table 1).

In order to show regional disparities clearly, Figs. 5 and 6 present the status of Slovak regions in terms of average values of SMR for IHD during the period 1996–2013, both for men and women. The worst values were observed in the Eastern Slovakia (KI, PV) and the best results were associated with the Western Slovakia (NI, BL). It is interesting that the value of SMR were ranked downward in the southern regions, from Eastern Slovakia to Western Slovakia. This likely relates to declining living standards away from East to the West. Standards of living can be expressed, for example, in terms of the unemployment rate that reached the value of 19.35 % (PI), 17.23 % (KI), 18.26 % (BC), 12.52 % (NI) and 6.17 % (BL) in 2013. Northern regions of Central and Western Slovakia (ZI, TC, TA) were characterised by an average unemployment rate of 10.8 %. There is a very strong positive statistical correlation (*r* = 0.93) between the SMR for IHD and unemployment rates across the regions.

**Fig. 7:**
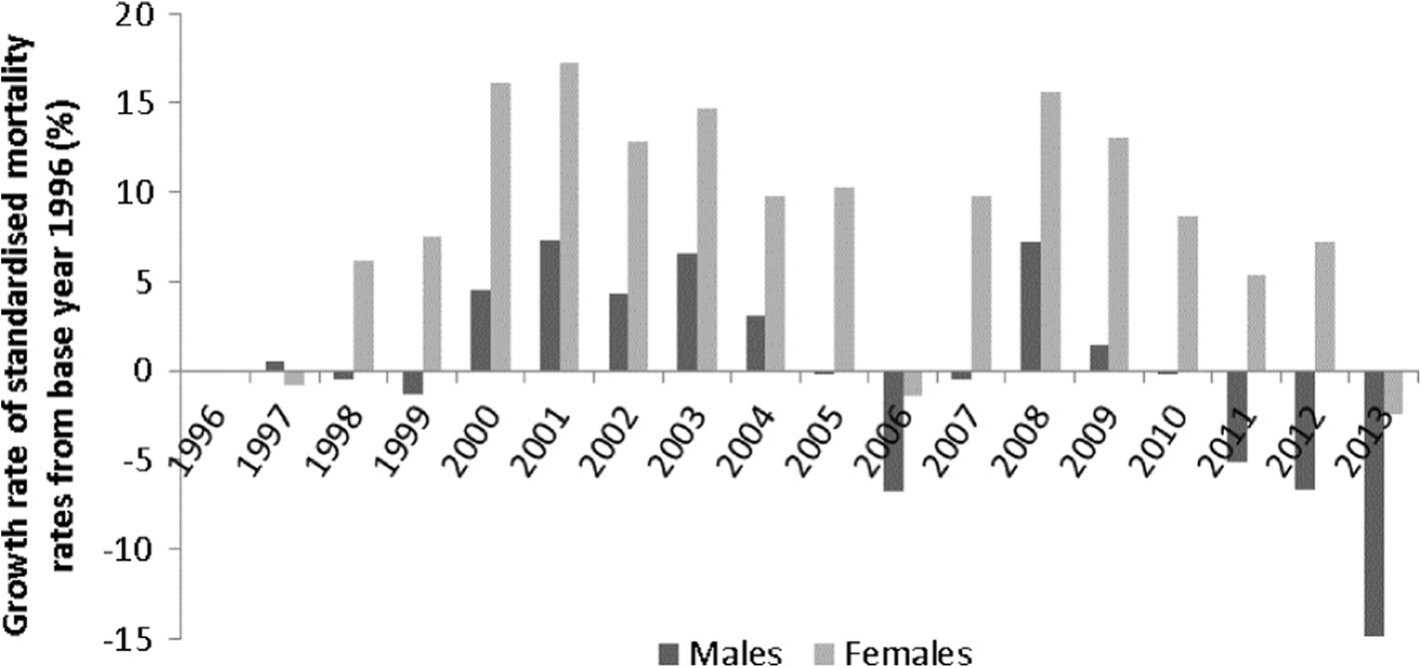
Growth rate of the standardised mortality rates for ischemic heart diseases, relative differences from the average standardised mortality rates in a base 1996 in Slovakia, 1996-2013, both sexes

Growth rate of SMR, relative differences from the average SMR in base year 1996, mostly showed worsening performance, both for males and females. These are the most visible to the average values of SR in 1998–2005 and 2007–2012 for women, and in 1997, 2000–2004, 2008 for men. In other years, the growth rate was lower compared to the 1996 (Fig. 7). According to Fig. 7, in 2013 the SMR were the closest to the level of SMR in 1996 for females. This indicates the existence of negative factors affecting the SMR for women in those years. The results were slightly better for males. The worst results were recorded in 2000–2004 and 2008. Significant decline in growth rate, in relation to 1996, occurred in 2006 and from 2011 onwards had a downward trend. In 2013, there was the biggest drop in SMR (-14.85 %) in relation to a base year 1996.

### The relationship between standardised mortality rates for IHD and income inequality in Slovak regions

Results of SMR for IHD in regions were confronted with indicators of income in these regions over the past 2013. Among the indicators of income inequality were included Gini coefficient, S80/S20, at risk of poverty threshold (60 % of national median equivalised disposable income), mean equivalised net income per household (EUR/month), unemployment rate in %. The correlation matrix reflects the level of dependence between SMR and each of the income indicators (Table 3).

**Table 3:** Correlation matrix of income indicators and standardised mortality rates for ischemic heart diseases in Slovakia, 2013

	SMR	S80/S20	At risk of poverty	Gini	Mean equivalised net income	Unemployment rate
SMR	1,00					
S80/S20	0,26	1,00				
At risk of poverty	0,79	0,70	1,00			
Gini	-0,04	0,95	0,45	1,00		
Mean equivalised net income	-0,84	-0,17	-0,81	0,12	1,00	
Unemployment rate	0,93	0,37	0,81	0,06	-0,82	1,00

*SMR* Standardised Mortality Rates

**Fig. 8:**
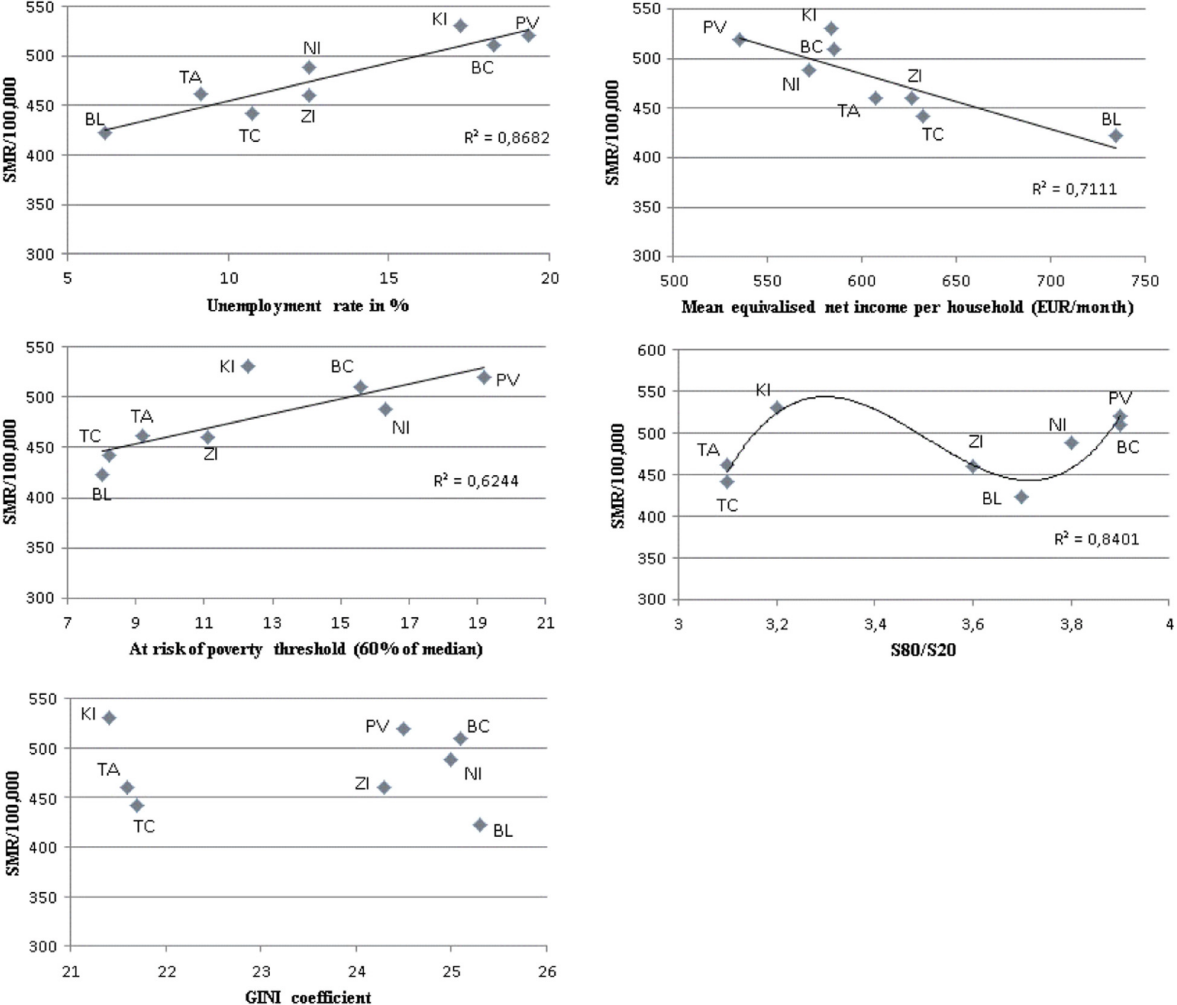
The relationship between standardised mortality rates for ischemic heart diseases and income indicators in Slovak regions, 2013

Very high, almost perfect positive correlation, was demonstrated between SMR and the unemployment rate (*r* = 0.93). Also very high negative correlation of equivalent net household income (*r* = -0.84) with SMR was found out. Among mentioned two income indicators also exists high degree of negative correlation (*r* = -0.82) (Table 3). Regions KI, PV, BC had the highest unemployment rates, and likewise the lowest equivalent net income which is also found in NI. BL held the reverse position (Fig. 8). High dependence (*r* = 0.79) was showed also between SMR and the rate of poverty, as a share of persons with an equivalent disposable income below 60 % of the national median. According to EU-SILC [31], median equivalent disposable income was 567 EUR/month per person in Slovakia. High negative correlation (*r* = -0.81) was observed between the poverty rate and equivalent disposable income. At risk of poverty threshold was the highest in the regions PV, NI, BC, and KI. Very low dependence (*r* = 0.26) was between SMR and S80/S20, according to which the lowest income inequality were in regions TC, TA, KI, and the highest in NI, BC, PV. Between SMR and Gini coefficient, there was no dependence (*r* = -0.04). Conversely, very high dependence (*r* = 0.95) showed between the GINI and S80/S20. Gini coefficient indicated the regions KI, TA, TC with the lowest income inequality (Table 3, Fig. 8).

According to Fig. 8, we can identify two main clusters of regions: regions with poorer levels of income indicators (KI, PV, BC, NI) and regions with better values (BL, TC, TA, ZI).

Regarding the effect (β coefficient) and significance (*p*-value) of each indicator on SMR (Fig. 8), three simple linear regressions were carried out as we expected high multicollinearity between income indicators. The dependent variable was SMR and the independent variables were individual income indicators. According to Fig. 8, there is non-linear polynomial regression between SMR and S80/S20. As we expected, the highest effect on SMR was observed in the unemployment rate (β = 7.75; 95 % confidence interval: 4.74 to 4.73), the poverty rate had a lower weight (β = 7.39; 95 % confidence interval: 1.66 to 13.12) and the equivalent disposable income reported the lowest effect (β = -0.55; 95 % confidence interval: ((-0.90) to (-0.2)). Regression coefficients of the estimated variables are statistically significant, as *p*-value < 0.05 for all models. Index of determination explains 62.44–86.8 % of the total variability of the SMR (Table 4).

**Table 4:** Linear regressions between SMR and income indicators

Income indicators	Est. β	95 % confidence interval	t-statistics	*p*-value	R^2^
Unemployment rate	7,75	4,74 až 10,77	6,29	0,0008	0,87
Mean equivalised net income	-0,55	(-0,91) až (-0,2)	-3,84	0,0085	0,71
At risk of poverty	7,39	1,66 až 13,11	3,16	0,0196	0,62

*Est.* Estimator

Our assumptions about the positive linear relationship between SMR and the poverty rate, and between SMR and the unemployment rate were confirmed by the results of the correlation and regression analysis. The assumption of a positive linear relationship between SMR and S80/S20 was only partially confirmed, because the predominant part of model was better explained by the polynomial trend of the third degree. The assumption of the positive linear relationship between SMR and the Gini coefficient was not confirmed.

According to the low correlation between SMR and S80/S20, it is necessary to examine the share of the richest to the poorest people in a broader sense in Slovakia, for example, interdecile range in which the inequality of income distribution would be more pronounced.

Stated outputs contribute to the knowledge of the impact of different biosocial and socioeconomic characteristics on the development of regional disparities in health. By linking complex characteristics of health status and health behaviour of the inhabitants in different regions with macroeconomic data such as income, education, unemployment, etc., we get the valuable analytical platform that will be used not only for the process of setting active prevention programs, but also for the process of revising the Strategic Framework of the Slovak healthcare system. On the other hand, the transformation of society significantly contributed to widening health inequalities [34]. This is mainly a group of people that is severely disadvantaged in terms of access to health care and also from the perspective of individual responsibility for their health. Just further development of this specific socially excluded group of people can significantly affect the dynamics and scope of changes in mortality rates across the population of Slovakia.

## Conclusions

Mortality is characterised by a relatively great deal of inertia in its development, therefore it is not expected that described differences between the populations of Slovakia and other European countries will be diminished in the next couple of years. The progressive extinction of the previous generations burdened unfavorable development of health conditions, especially in the 70s and 80s, will also play a huge role. However, the key factor will be improving mortality ratios, especially in cardiovascular diseases.

In our paper, we presented evaluation of the development of mortality rates for IHD in Slovakia, in addition, our ambition was to quantify regional disparities and to analyse the development of mortality in relation to the income inequalities in the regions of Slovakia. SMR for IHD in Slovakia shows alarming values in comparison with EU-28. We recorded higher values of SMR nearly of 40 % for males, on the other hand, stronger rate of decline. The biggest differences between males and females occurred at the beginning of the examined period and the smallest gap was in the recent years. The growth rate of SMR in base year 1996 was higher for females than for males. The incidence of mortality from ischemic heart diseases has moved to higher ages over the years. People at the highest risk were at the age 70–84 in 1996–2001 and at the age 75–84 in 2002–2013.

Variability of SMR gradually declined among time periods, although the growth rate of SMR in base year 1996 showed a rather worsening character. The best seems to be the last year of 2013. During the explored period, the worst results of SMR were recorded in the region of KI and the best in NI for both sexes. Values of SMR were improving from Eastern Slovakia to Western Slovakia. However, the region of NI showed the highest variability of SMR, on the contrary, the lowest variability was typical for the region of KI.

As for females, the region of BL, TA and KI recorded the permanent percentage drop in SMR between time periods, on the other hand, the opposite trend was indicated in NI. Other regions had a changing rate of development. As for males, the region of BL, TA and KI recorded the same trend like for females, additionally the region of NI and BC. Other regions had a changing rate of development.

We also investigated the relationship between the SMR and income indicators across regions. Very high linear correlation was connected with the indicators: unemployment rate, at risk of poverty threshold (60 % of national median equivalised disposable income), mean equivalised net income per household. Conversely, a low correlation was found with the indicator S80/S20. There was no dependency between SMR and Gini. The two main clusters of regions were detected: regions with poorer levels of income indicators (KI, PV, BC, NI) and regions with better values (BL, TC, TA, ZI). Higher values of SMR were typical for the first group, conversely lower for the second group.

Using linear regression, we quantified the effect of income indicators on SMR. The highest positive effect was recorded for the unemployment rate and at risk of poverty threshold. An equivalent disposable income recorded the low, inversely proportional, influence. These three income indicators were statistically significant.

Testing the impact of other indicators expressing socioeconomic status of inhabitants living in the Slovak regions, such as education, employment, marital status, nationality, etc., could be the subject of further analyses. Only after identifying the mutual relations, we will able to set the health system to meet the needs and capabilities of individual groups of people.
